# Nonfunctioning Adrenal Incidentalomas: The Search for Subclinical Cardiac Alterations

**DOI:** 10.5935/abc.20180234

**Published:** 2018-11

**Authors:** José Luiz Barros Pena

**Affiliations:** 1 Pós-graduação Faculdade de Ciências Médicas de Minas Gerais, Belo Horizonte, MG - Brazil; 2 Hospital Felício Rocho, Belo Horizonte, MG - Brazil

**Keywords:** Incidental Findings, Diagnostic Imaging, Metabolic Syndrome, Hidrocortisone, Echocardiography Doppler, Cohort Studies

By definition, an adrenal incidentaloma (AI) is an asymptomatic adrenal mass detected incidentally on imaging examination not performed for suspected adrenal diseases.^1^ The prevalence of AI has been estimated to be as high as 4.2% upon evaluation of abdomen or thorax by computed tomography (CT) scans. In most cases (85%), AIs are nonfunctioning.

In this issue of *Arquivos Brasileiros de Cardiologia*, Sokmen et al.^2^ selected patients (Pts) following the European Society of Endocrinology Clinical Practice Guideline in collaboration with the European Network for the Study of Adrenal Tumors. After confirming the presence of an adrenal adenoma through imaging tests, computed tomography, or magnetic resonance imaging, they excluded Cushing Syndrome through 1-mg dexamethasone suppression test (DST), pheochromocytoma by urinary fractionated metanephrine test, and primary aldosteronism.^3^

Patients with nonfunctioning AIs may have mild hypercortisolism, reduced insulin sensitivity, and increased blood pressure levels when compared to controls.^4^ Previous studies have demonstrated that insulin resistance, hypertension, dyslipidemia, fatty liver disease, and metabolic syndrome were identified in Pts with nonfunctioning AIs.^4^^,^^5^ Current understanding is that nonfunctioning AIs may secrete small or undetectable amounts of cortisol that may cause mild systemic changes.^4^ Adequate management and follow up of these Pts has yet to be established. Morphological and functional cardiac alterations have been insufficiently reported for this particular group.^4^^-^^6^

Echocardiography seems to be to most versatile noninvasive imaging technique to assess volumes, ejection fraction, myocardial mass index, diastolic function, right ventricular (RV) function, hemodynamics, and valvular regurgitation.^7^ This study by Sokmen et al. is important as it measures atrial electromechanical delay (EMD) by utilizing tissue Doppler echocardiography (TDE). EMD has proven to be valuable in predicting new onset or recurrence of atrial fibrillation.^7^ Atrial fibrillation is one of the most common arrhythmias in clinical practice, associated with significant mortality, morbidity, and thromboembolic events. Several publications confirm the value of tissue Doppler when measuring EMD parameters to identify Pts susceptible to this condition.^8^ In this particular AI group, atrial conduction times were measured.

Both inter-atrial EMD and intra-atrial EMD were higher in the nonfunctioning AI group compared to controls. According to the authors, this is the first time these abnormal measurements have been demonstrated in the literature. The authors found some indirect evidence of autonomous adrenal secretion and identified that post-DST cortisol level was an important predictor of intra-atrial EMD. They deduced that the increase of post-DST cortisol level by 1 µg/dL lengthened intra-atrial EMD by 4.752 ms.

The anatomical and morphological findings demonstrated that diastolic thickness of the interventricular septum, posterior wall, and left ventricular (LV) mass index were significantly higher and pulmonary acceleration time significantly lower in the nonfunctioning AI group compared to the control group. Tissue Doppler Em/Am measurements in LV lateral, septal, global, and RV were significantly decreased in the nonfunctioning AI group, confirming tissue Doppler as a tool of both global and regional functions.^9^

The results would have been more robust if these Pts had been followed up for a longer period. The deformation indices (strain/strain rate) of both ventricles and atria could add important data to the study, as they are superior to tissue Doppler in detecting subclinical abnormalities. Notwithstanding, the authors demonstrated that indeed subclinical cardiac involvement exists in nonfunctioning AI Pts. Therefore, as they show increased cardiovascular risk, they should be followed up more frequently.
